# Does the Experience of Remembering Differentially Influence the Factual Accuracy of Recognition, and Confidence in Its Accuracy?

**DOI:** 10.5334/joc.477

**Published:** 2026-01-07

**Authors:** Phyllis Margaret Windsor, Benjamin R. Dering, David I. Donaldson

**Affiliations:** 1 Psychology, Faculty of Natural Sciences, University of Stirling, Stirling, FK9 4LA, Scotland, UK; 2 School of Psychology and Neuroscience, University of St Andrews, St Mary's Quad, South St, St Andrews, KY16 9JP, Scotland, UK

**Keywords:** accuracy, confidence, recognition, source memory, vividness

## Abstract

Remembering is typically viewed as unreliable and prone to errors, whereas highly confident recognition memory is often believed to be highly reliable and associated with high recognition accuracy. We evaluated these beliefs using memory for photographs of natural scenes in two studies: recognition memory to examine picture similarity effects in a 2-alternative forced-choice measure, and source memory to examine picture-location associations with a continuous retrieval accuracy measure. Additionally, we assessed the experience of remembering and its influence on judgments of confidence and memory accuracy. High confidence remembering was associated with high accuracy when perceptually or mnemonically similar lures were presented in the item recognition task. However, an association between high confidence and high accuracy was also seen in the absence of remembering for mnemonically similar lures. The confidence-accuracy inversion in the picture similarity task is speculated due to confidently (mis)remembering a similar picture stored in memory. Based on analyses of participant and trial level data, in both studies memory quality was strongly associated with confidence. Importantly, remembering moderated the association between recognition accuracy and confidence judgments, differentially influencing confidence more than it influenced accuracy. Memory quality moderated the association between source accuracy and confidence, the relationship being stronger for images remembered vividly. Our findings have implications for accounts of vividness, confidence, episodic memory, and eyewitness testimony. High confidence recognition may not in all cases reliably imply high accuracy. Highly vivid memories, confidently recollected, may not always be factually accurate.

## Introduction

Despite the inherent fragility of memory, researchers have asserted that under normal circumstances there are no established grounds for considering memory unreliable (Brewin, Andrews & Mickes, 2020), thus we should have confidence in the accuracy of our memories. Consequently, in applied settings such as eyewitness testimony, recognition memory is generally considered reliable, reflected in the fact that remembering with high confidence is associated with high recognition accuracy (e.g., Brewer & Wells, 2006). Nonetheless, researchers also acknowledge that eyewitnesses may sometimes be highly confident but inaccurate, and the use of predictive variables, e.g., time to decision, and responses based on familiarity, have been suggested (Grabman, Dobolyi, Berelovich & Dodson, 2019). Furthermore, a large body of work has demonstrated that confidence can be readily influenced and may sometimes provide a limited indication of accuracy (e.g., Leippe, 1995; Smith, Smalarz, Ditchfield & Ayala, 2021). Here, therefore, using two different memory paradigms, we re-assess the belief that remembering with high confidence is a dependable guide to memory’s accuracy.

The association between accuracy and confidence in recognition is classically tested using an old or new (item recognition) task. At test, participants are required either to recognise or remember stimuli previously shown at study. Additional information about the memory experience can also be provided, for example, by asking participants to subsequently report confidence in the accuracy of their decisions, or to provide judgments of ‘remembering’ or ‘knowing’ that reflect different subjective experiences during retrieval (Gardiner, 1988; Rajaram, 1993; Tulving, 1985). In a standard old or new recognition task, correct compared to incorrect recognition decisions (e.g., Sporer, Penrod, Read & Cutler, 1995), and remembering compared to knowing (Dunn, 2004; Gardiner, 1988; Tulving, 1985), are both associated with significantly higher ratings of confidence.

Here we consider whether the accuracy of memory or the experience of remembering (and its quality) is the more important for determining the confidence an individual has that their memory is correct. Based on data from a variety of experimental studies, we know that multiple factors may be responsible for judgments of memory confidence (Brewer & Sampaio, 2012), leading to dissociations of confidence and accuracy. Confidence can also be subject to individual differences (e.g., Ais, Zylberberg, Barttfeld & Sigman, 2016). Furthermore, participants may base their confidence on cues other than those that determine recognition accuracy (Busey, Tunnicliff, Loftus & Loftus, 2000; Zawadzka, Higham & Hanczakowski, 2017).

More broadly, metacognitive assessments of confidence are considered inferential. For example, they may be based on fluency (how quickly an answer is retrieved from memory), task difficulty, consensuality, or familiarity, as well as the quality of the recollective experience (Chua, Hannula & Ranganath, 2012; Kelley & Lindsay, 1993; Koriat, 2008; Kurdi, Diaz, Wilmuth, Friedman & Banaji, 2018; Olsson, 2000; Robinson, Johnson & Robertson, 2000). What studies to date have failed to reveal, however, is whether such cues affect confidence and accuracy in the same way, considering that other factors (such as the influence of prototypes, or sleep deprivation) have been shown to differentially affect confidence (Blagrove & Akehurst, 2000; Kerrén, Zhao & Griffiths, 2024). Of particular relevance here is the finding that the subjective experience of remembering occurring during a recognition task is considered to be a major factor in generating confidence judgments (Jersakova, Allen, Booth, Souchay & O’Conner, 2017), yet it remains unclear whether the experience of remembering equally affects the accuracy of recognition and our confidence in its accuracy.

Additionally, here we also ask whether the quality of retrieved memories is important for judgments of confidence. Briefly, it is accepted that if we consider a stimulus vivid, it is later recognised more accurately (Jacoby, Baker & Brooks, 1989; Spence, Wong, Rusan & Rasteger, 2006; Wichmann, Sharpe & Gegenfurtner, 2002). Additionally, memory quality can be suggested by how vividly a memory is perceived. If we refer to a memory as vivid, we are more likely to feel we are reliving the experience (Buchsbaum, Lemire-Rodger, Fang & Abdi, 2012; Rubin, Deffler & Umanath, 2019). Equally, the experience of recollection can be enhanced for emotional events, where vividness is defined as internal, because the event had personal significance which provoked an emotional reaction (Cooper, Kensinger & Ritchey, 2019; Ford & Kensinger, 2017; Habermas, Diel & Welzer 2013; Kinugawa, Schumm, Pollina, Depre, Jungbluth… & Dere, 2013; Phelps & Sharot, 2008), or because the stimulus triggered spontaneous recollection of a personal memory (Gardiner, Ramponi & Richardson-Klavehn, 1998).

There is ample evidence that vividness is associated with confidence in a memory’s accuracy. To the lay audience, for example, personal memories deemed vivid are more likely to be confidently believed. In addition, studies involving flashbulb memories (i.e., memories of historically significant world events) often report that such memories are detailed, long-lasting, and vivid (Brown & Kulik, 1977; Edery-Halpern & Nachson, 2004; Luminet & Spijkerman, 2017; Pillemer, Goldsmith, Panter & White, 1988). Vivid memories may not always be accurate, however, and other researchers have shown the contextual details of flashbulb memories can sometimes be incorrect or lacking (Stone, Luminet & Takahashi, 2015; Talarico, Kraha, Self & Boals, 2019; Talarico & Rubin, 2003; 2007). Equally, when asking whether remembering and its quality (i.e., vividness) might be one of many factors which underlie dissociations between confidence and accuracy, the current literature shows varying degrees of association between memory vividness and memory accuracy (e.g., Habermas & Diel, 2013; Richter, Cooper, Bays & Simons, 2016). Consequently, in addition to studying the experience of remembering and its influence on both confidence and memory accuracy, here we also assess how these relationships may be affected by variability in memory quality (i.e., remembered vividness).

## Study 1 – Recognition Accuracy, Remembering and Confidence

### Introduction

In Study 1, we considered the effect of remembering and its quality on both recognition accuracy and confidence in the recognition decision. We asked participants to study a set of pictures of natural scenes and then complete a recognition test with recollection (i.e., remember), confidence, and vividness ratings taken after each test trial. To measure memory, we chose a two-alternative forced-choice (2-AFC) old or new picture similarity recognition task (Tulving, 1981). Although items are shown separately at study, the two alternatives (old and new, target and lure respectively) are presented simultaneously at test, with the participant having to discriminate between them whilst information from both stimuli is available. Apart from indicating which of the test pictures was old, in the original task participants also rated confidence in their decision on a three-point scale. Tulving’s picture similarity experiment manipulated stimuli at test to make targets and lures similar, either perceptually or mnemonically, to produce a dissociation between confidence and accuracy and to test whether recognition accuracy was inversely related to the similarity between old and new test items.

This paradigm offers good replicability in producing a confidence-accuracy inversion between picture pairs shown at test with perceptual and mnemonic similarity (Dobbins, Kroll & Liu, 1998; Fandakova, Johnson & Ghetti, 2021; Heathcote, Freeman, Etherington, Tonkin & Bora, 2009; Hembacher & Ghetti, 2017; Zawadzka et al., 2017), with perceptually similar test-pairs providing more accurate recognition than mnemonically similar test-pairs (which are dissimilar to the target, but perceptually similar to a non-target picture shown at study). If the lure and the target are highly similar, however, participants are more accurate but less confident. Tulving explained this dissociation as representing two types of similarity relations in forced-choice recognition memory. When pictures are perceptually similar, the retrieval process to determine which picture was old is more elaborate and results in higher accuracy than when the pictures are perceptually dissimilar – and memory of the picture that was similar to the lure was relied on to make the decision.

Tulving’s demonstration of the confidence-accuracy inversion is important because it highlights conditions in which confidence and accuracy can be dissociated – at least when the nature of lures is manipulated. Findings from a study by Busey et al., (2000) using memory for faces also suggests confidence need not always reflect accuracy. Using an old-new face recognition experiment Busey and colleagues found that brightening face images at test (shown dim at study) increased subjective confidence without influencing accuracy, suggesting that the consequence of both study and test conditions may lead to confidence being affected over and above accuracy. Similarly, other researchers have demonstrated that confident false recognition of deceptive lures can lead to illusions of memory accuracy (Roediger & McDermott, 1995; Tulving, 1981; Weigl, Pham, Mecklinger & Rosburg, 2020).

The impact that varying the nature of lures has on the relationship between confidence and accuracy is of particular importance in relation to the eyewitness field, where recognition of a suspect is commonly assessed using either a task where pictures of faces are shown singly, or as one photograph in a set of photographs (i.e., representing an N-AFC task, where multiple images are shown together equivalent to an identity parade/lineup). The relative merits of these two tasks has been debated at length in the wider literature (e.g., see Wixted & Mickes, 2015). In a lineup, eyewitnesses are claimed to be less accurate (more prone to false recognition) if the suspect and fillers are similar. That is, the more similar lures or innocent people are to targets (suspects), the more confident false identification (false recognition) is likely to occur (cf. DeSoto & Roediger, 2014; Grabman et al., 2019; Roediger & DeSoto, 2014). In other research, however, correct recognition has been shown to be lower, and incorrect recognition higher, when a lure substituted for a target face picture was of high similarity (Brewer & Wells, 2006). In this case, therefore, results suggest that testing highly similar faces in a lineup is more likely to cause recognition errors.

Regardless, Colloff and Wixted (2020) make a case for “fair” simultaneous lineups, where all members match the description of the suspect to provide eyewitnesses with the opportunity to compare features to boost identification accuracy. Conversely, in an “unfair” simultaneous lineup the suspect stands out because they are more similar to the participant’s memory of the real perpetrator than are the other lineup members. From this perspective the confidence-accuracy inversion effect largely occurs when memory is poor (e.g., not based on remembering), or testing procedures are biased (e.g., by inappropriate lures), and when appropriate testing conditions are maintained high confidence is strongly predictive of high accuracy. As we show here, however, confidence in the accuracy of recognition is also affected by the quality of memory and the experience of remembering – not just its factual accuracy.

### Methods

#### Recruitment

Participants were psychology undergraduates attending the University of Stirling, Scotland U.K., aged 18–37 years with normal or corrected-to-normal vision and no history of abnormal colour perception, who took part for mandatory course credits. The studies were approved by the University of Stirling’s General University Ethics Panel (GUEP). Full details of the online recruitment protocol is provided in Supplementary file 1: Appendix. Study 1, Recruitment and online instructions to participants. We recruited 70 participants of mean age 21 years (*S.D*. 5 years); 56 identified as female. A sample size of 60 participants provides sufficient statistical power (0.78) for an experiment designed to determine, for the same subjects, if three data sets (i.e., test picture-pair conditions) are significantly different from each other at 5% significance level (two-tailed), using one-way ANOVA.

##### Stimuli

Real-world colour natural scene photographs were used for targets and lures. We selected images from four distinct categories of natural scenes: flowers, green landscapes and trees, mountains, and water, because previous evidence suggests diverse types of natural scenes are associated with equivalent levels of recognition accuracy (Wichmann et al., 2002). Because the picture similarity task involves perceptually similar and dissimilar pictures, we required a large number of images. Suitable images were sourced and downloaded from two online psychology image databases, the McGill Calibrated Color Image Database (Olmos & Kingdom, 2004), and the U-Penn calibrated natural images of the Okavango Delta of Botswana (Tkačik, Garrigan, Ratliff, Milčinski, Klein, Seyfarth… & Balasubramanian, 2011). Because of the difficulty in downloading sufficient suitable images for the categories required, further photographs of natural scenes were selected from the corresponding author’s personal database of natural scene colour photographs from the UK and elsewhere. To ensure none of the images were obviously less visually vivid, contrast and colour saturation were adjusted if required. All photographs used in Study 1 can be viewed online as detailed in the data accessibility statement. To minimise content that would potentially bias recognition accuracy or image memorability, we avoided photographs including people or animals, and those featuring incongruent objects or man-made structures such as famous buildings (Evans & Baddeley, 2018; Hunt, 2013; Santangelo, Di Francesco, Mastroberardino & Macaluso, 2015; VanArsdall, Nairne, Pandeirada & Cogdill, 2017; Wichmann et al., 2002). Faces with positive and negative expressions of emotion and pictures depicting positive and negative events are considered to be emotionally-valenced (e.g., Keightley, Winocur, Graham, Mayberg, Hevenor & Grady, 2003). We deliberately did not include images with potential emotionally evocative features (i.e., including people with emotional expressions, or animals), as such scenes can influence recognition relative to neutral scenes (e.g., Keightley, Chiew, Anderson & Grady, 2011), and can influence both memory accuracy and confidence (Phelps & Sharot, 2008). Nonetheless, as would be the case for any images, we could not rule out idiosyncratic emotional interpretation of the images arising from participants liking or disliking the picture, or from images provoking a pleasant or unpleasant personal memory.

#### Procedures

##### Study sequence

**Figure 1:**
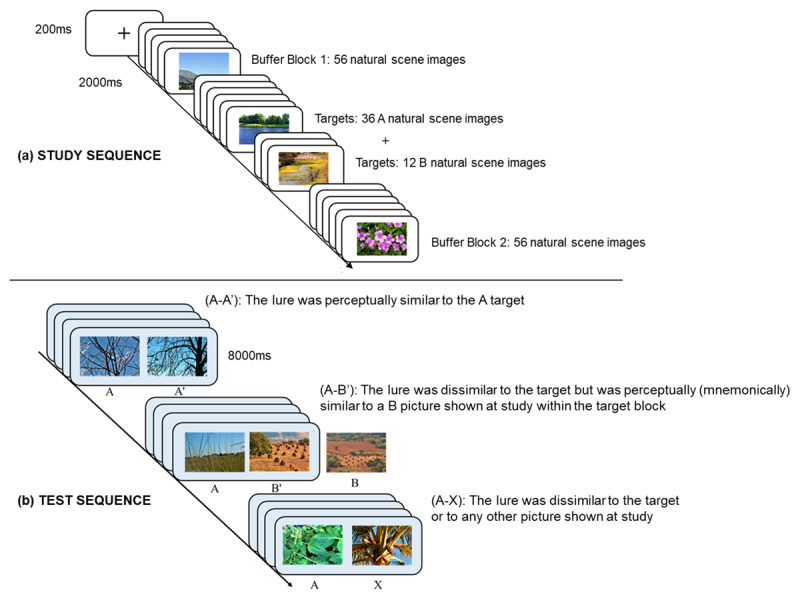
Study 1: protocol for the 2-AFC picture similarity recognition memory task. **(a)** Study sequence: 160 pictures were shown in random order within both buffer and target blocks, each image was displayed for 2000 ms following a fixation cross (200 ms). Targets were not identified as such to participants, who were instructed to try to pay attention to all the images; **(b)** Test sequence: example picture-pairs used in the test phase (all pairs were shown in random order). Note, for illustration purposes the target A picture is shown on the left and the lure on the right. For comparison, the B picture shown in the target block is also illustrated (not shown at test). Pictures illustrated are all from the category of green landscapes and trees.

The experiment was carried out online using the participant’s own tablet or PC (but not a smart phone). Duplicating Tulving’s (1981) original task, 196 different natural scene photographs were used: 160 photographs were arranged into three blocks, shown sequentially in a fixed order with no gap between the blocks. First, a buffer block of 56 photographs, which were not used for the test sequence; second, a target block of 48 photographs (not identified as such to the participants) consisting of 36 A targets and 12 B photographs (which were not shown again but were similar to the B’ lures shown at test); third, a second buffer block of 56 photographs, which were not used for the test sequence (Figure 1a). Following a fixation cross (200 ms), each picture was displayed for 2000 ms in a continuous sequence. The sequencing of photographs within each study block was randomised for each participant by the online platform. The additional 36 images that were not shown at study were used as lures, paired with the 36 A (old) targets shown in the test sequence. To ensure a timed break between study and test phases, after viewing all images in the study phase participants were asked to look at a “Where’s Wally” picture for two minutes before they were advanced to the test phase.

##### Test sequence

At test, the 36 A targets were shown again. each with a lure (a new previously unseen picture) as a test picture-pair. The test picture-pairs were randomly displayed in each of three conditions: (A-A′), the lure was perceptually similar to the A target; (A-B′), the lure was dissimilar to the target but was perceptually (mnemonically) similar to one of the 12 B pictures shown at study within the target block; and (A-X), the lure was dissimilar to the target or to any other picture shown at study. All test picture-pairs were matched and counterbalanced for natural scenes subject category (three of the 12 test-pairs in each of the three conditions were taken from one of the four natural scenes subject categories (Figure 1b). All participants viewed the same 12 test picture-pairs displayed for 6000 ms in random sequence in each of the three conditions. The target picture was pseudo-randomized to appear either on the left- or right-hand side of the screen. Full details of the image selection for test picture-pairs used in Study 1 are provided in Supplementary file 2: Appendix. Study 1: Picture similarity criteria.

##### Reaction time

Output from the testing platform included time (ms) for the participant to make their recognition decision. In addition to enabling exclusion of trials that were timed out, reaction times could be evaluated for each test picture-pair condition.

##### Behavioural data

Participants’ behavioural responses during testing were recorded, starting with the time (ms) participants took to make their recognition decision (enabling exclusion of trials that took longer than the maximum time allowed of 6000 ms). On all test trials, once a decision had been made (i.e., the participant had selected one of the picture-pair as being old) behavioural data were collected from participants in a fixed order: vividness, confidence, and remembering. Vividness (‘how vividly did you remember the picture?’) and confidence (‘how confident are you that you remembered the picture correctly?’) ratings were both assessed using a 0–100 sliding scale and were therefore asked one after the other, with the ‘yes or no’ remember response asked last. The vividness judgment was presented first to ensure that vividness ratings related to the memory retrieval decision; equally, it was considered appropriate to rate memory vividness before assessing confidence, as accuracy is thought to influence confidence.

Based on previous reported studies (e.g., Gardiner, Ramponi & Richardson-Klavehn, 2002), participants were instructed to answer yes or no to whether they had remembered the picture recognised as old, so as not to force participants to answer Know if a stimulus was not remembered. Additionally, before leaving the experiment, participants completed a short online checkbox questionnaire based on how well they complied with the Remember yes or no instructions. Full details of the task instructions are provided in Supplementary file 1: Appendix. Study 1, Recruitment and online instructions to participants.

#### Data Analysis

Data were analysed using the Statistical Package for Social Scientists (SPSS Statistics for Windows, Version 29.0, released 2022. Armonk, NY: IBM Corp.), and jamovi {(The jamovi project (2021). jamovi (Version 1.6) [Computer Software], retrieved from https://www.jamovi.org)}. Testing for moderation was carried out using the PROCESS macro for SPSS (Hayes & Scharkow, 2013; Hayes, Montoya & Rockwood, 2017).

### Results

#### Participants

To ensure data quality accuracy (proportion of correct responses) was analysed for each participant relative to the mean [*M* (*S.E*.) 0.62 (0.01)]. One outlier with a response accuracy of 0.28 was excluded from the subsequent data analysis: of the 36 trials carried out, 17 were timed out, all trials were judged as remembered. Data from five other participants who judged all 36 trials as remembered were excluded, as it was considered they had either misunderstood or not carried out the instructions. The final data set comprised 64 participants, completing a total of 2194 trials, 110 timed-out trials (4.8%) were excluded from the analysis (recognition decisions were not recorded on timed out trials).

#### Variables of interest

**Figure 2:**
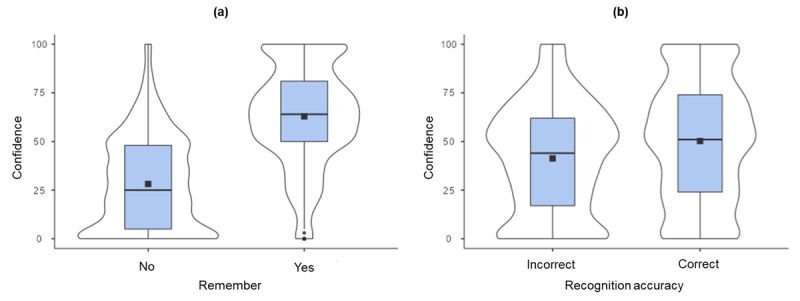
Study 1: illustrating the distribution of trial level confidence ratings **(a)** by the remember response (no or yes); **(b)** by recognition accuracy (incorrect or correct). Box plots show median values as horizontal black bands. The associated violin plots illustrate the distribution of responses, along with mean values, shown as black squares. A straight line between the mean values is equivalent to a point biserial plot.

As can be seen in Figure 2a, confidence was significantly higher when the recognition decision was associated with the experience of remembering compared to the absence of remembering [median = 64 and 25 respectively; *M* (*S.E*.) = 62.9 (0.74) and 28.2 (0.76) respectively; *n* = 1179 and 1015 trials respectively]. The substantial number of individual trials analysed makes even tiny differences result in significant *p*-values. Here, therefore, we report the unstandardised beta coefficient (*B*), produced by regression analysis when the analysis is performed on original, unstandardised variables. Interpretation of effect sizes were related to the *R^2^* change, taken as (0.01) 1% = weak, (0.09) 9% = moderate, and (0.25) 25% = strong (Cohen, 1992; Taylor, 1990). Trial level ANOVA revealed a significant difference [*F* (1, 2192) = 1071, *p* < 0.001], with a strong effect size (*B* = 34.74; *R^2^* change = 32.8%). Figure 2b illustrates confidence was also significantly higher when the recognition decision was correct compared to incorrect [median = 51 and 44 respectively; *M* (*S.E*.) = 50.2 (0.84) and 41.3 (0.97) respectively; *n* = 1360 and 834 trials respectively]. Analysis again revealed a reliable effect [*F* (1, 2192) = 46.0, *p* < 0.001], but critically, in this case the effect size was weak (*B* = 8.93; *R^2^* change = 2.1%).

Variables of interest were compared by test-pair condition, revealing a confidence-accuracy dissociation between perceptually and mnemonically similar conditions (see Table 1). Overall differences in confidence and accuracy were followed up using pair-wise independent samples *t*-tests (two-sided), revealing that confidence was higher for the mnemonically similar A–B’ condition compared to the perceptually similar A–A’ condition (*p* = 0.01; *d* = 0.14), whereas accuracy (proportion correct) was not significantly different (*p* = 0.12). Neither the proportion of trials remembered, nor reported memory vividness, differed significantly across conditions. Participants took significantly longer to decide which of the pictures was old in the A–A’ condition compared to both A–B’ (*p* = 0.002; *d* = 0.16) and A–X (*p* = < 0.001; *d* = 0.2) conditions.

**Table 1:** Study 1: variables of interest [confidence, recognition accuracy (proportion correct), proportion remembered, vividness, and reaction time] by test-pair condition.


TEST PICTURE PAIR CONDITION (*n*)	CONFIDENCE	PROPORTION CORRECT	PROPORTIONREMEMBERED	VIVIDNESS	REACTION TIME, (ms)

A–A’ (724)	44.6 (1.09)	.63 (0.02)	0.52 (0.02)	50.05 (1.07)	3178.3 (48.74)

A–B’ (731)	48.65 (1.13)	.59 (0.02)	0.56 (0.02)	52.34 (1.08)	2974.9 (45.74)

A–X (739)	47.21 (0.65)	.65 (0.02)	0.53 (0.02)	50.58 (1.08)	2923.0 (44.28)

ANOVA	*F* (2, 2191) 3.36; *p* = 0.04, η^2^ = 0.003	*F* (2, 2191) 3.06; *p* = 0.05, η^2^ = 0.003	*F* (2, 2191) 1.82; *p* = 0.16	*F* (2,2191) 1.24, *p* = 0.29	*F* (2, 2191) 8.48; *p* < 0.001, η^2^ = 0.008


Note. Results are compared by one-way ANOVA. Effect sizes are represented by the partial η^2^ statistic, where η^2^ = 0.01 indicates a small, η^2^ = 0.06 a moderate, and η^2^ = 0 .14 a large effect.

#### Confidence Accuracy Characteristic (CAC) Analysis

We next evaluated our data using a calibration approach, i.e., using Confidence Accuracy Characteristic (CAC) analysis. Metacognitive calibration measures the accuracy associated with individual levels of confidence (e.g., how accurate are responses given a 100% confidence rating). Recognition accuracy, dependent on recognition of the target item as old (Zawadzka et al., 2017), is represented using calibration curves, calculated by dividing metacognitive confidence judgments into bins, e.g., 0–10, 11–20… and determining the average accuracy (proportion correct) for each bin. Plotting the results is equivalent to a calibration (Palmer, Brewer, Weber & Nagesh, 2013). For assessments where the outcome is binary (hit or miss, yes or no, correct or incorrect), this method of calibration equates with a confidence accuracy calculation. Proposed as an alternative to Receiver Operator Characteristics (ROC) curve analysis, CAC plots are considered to be more intuitive, for example as a way of relating identification accuracy to eyewitness confidence (Mickes, 2015), mapping participants’ average accuracy to their averaged or binned metacognitive judgments of confidence (assuming that participants can reliably rate their psychological experience on to the scale used (cf. Rhodes, 2019).

A perfect linear CAC relationship would be characterized by 100% averaged accuracy for those witnesses who were 100% confident, 90% accuracy for witnesses who were 90% confident, and so on. Eyewitness researchers (e.g., Brewer & Wells, 2006; Vuorre & Metcalfe, 2022; Wixted & Wells, 2017) have increasingly become more concerned with calibration than with measures of metacognitive resolution (indicating how individuals can judge their own accuracy using confidence ratings). In earlier research, for example, Bornstein and Zickafoose (1999) plotted average predicted confidence against accuracy for responses to both eyewitness and general knowledge questions.

More recently, Tekin, DeSoto, Wixted and Roediger (2021) demonstrated how a CAC plot can be adjusted for use in a binary old or new discrimination task, as a direct alternative to ROC curve analysis. These authors used two different methods of calculation to create the plot: either item-based accuracy, where proportion correct = hits ÷ (hits + misses), or response-based accuracy, where proportion correct = hits ÷ (hits + false alarms). In either case findings revealed that confidence was always highly related to accuracy, with both related and unrelated lures. Here, our 2-AFC paradigm does not produce data for hits and misses, and thus we use the response-based accuracy method to create CAC plots, where proportion correct = hits ÷ [hits + false alarms (i.e., all responses)]. In addition, to avoid artificially biasing outcomes by tailoring the choice of bins, CAC plots were created using trial level data with confidence binned into quartiles (defined in a data driven fashion for each set of data analysed, e.g., values of 25, 50 & 75 for a median value of confidence = 50, dividing the number of data points into quartiles of more-or-less equal size), allowing comparison of the averaged values of recognition accuracy (proportion correct) for each level of binned confidence.

**Figure 3:**
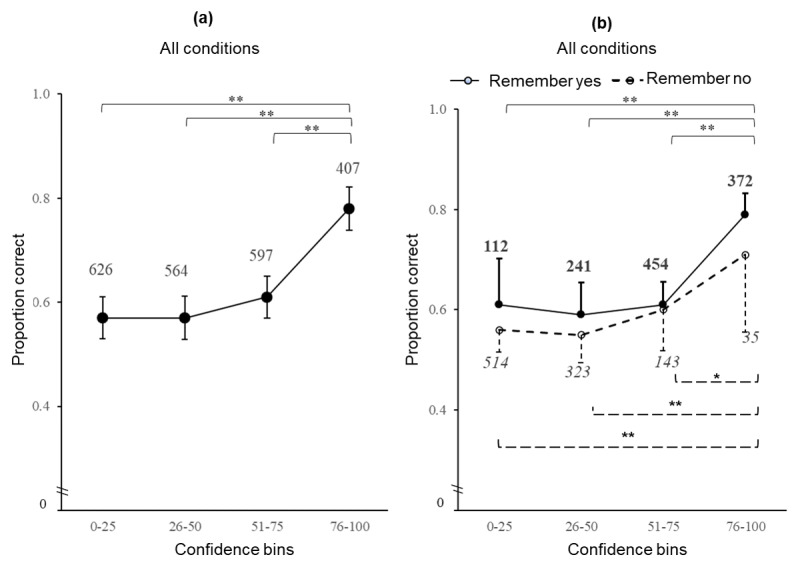
Study 1: CAC plots of trial level data across all test-pair conditions, illustrating recognition accuracy (mean proportion correct) by confidence (binned in quartiles): **(a)** overall data; **(b)** the overall data split by the experience of remembering. [(** *p* < 0.001), (* *p* < 0.01). Error bars represent 95% *C.I*. – numbers represent trials assessed within each bin of confidence – only positive or negative bars are shown in Figure 3b for clarity; numbers represent trials assessed within each bin of confidence – bold figures represent remembered trials and italic figures represent trials associated with not remembering].

As illustrated in Figure 3a, when examined across all data, CAC plots clearly demonstrate that as accuracy increases, higher confidence is reported. Collapsed across remembered and not remembered responses, the overall proportion correct (i.e., accuracy) was significantly higher at high levels of confidence ≥ 76 (*M* = 0.78) than for all other bins [(51–75, 26–50, and 0–25; *M* = 0.61, 0.57 and 0.57 respectively), with all independent two-sided samples *t*-tests significant, *p* < 0.001, (Cohen’s *d* = 0.29, 0.39 and 0.37 respectively)]. The CAC plot also allowed us to separately examine the effect of remembering on recognition accuracy, illustrating for each level of binned confidence there was no significant difference in accuracy by remembering (Figure 3b). Notably, binned confidence was higher as the proportion of trials where remembering was reported increased. For responses associated with remembering, analysis confirmed accuracy was significantly higher at the highest bin of confidence ≥ 76 (*M* = 0.79) than for all other bins [(51–75, 26–50 and 0–25, *M* = 0.61, 0.59 and 0.61 respectively, independent two-sided samples *t*-tests all highly significant *p* < 0.001, (Cohen’s *d* = 0.38, 0.46 and 0.42 respectively)].

Furthermore, as accuracy increased, higher confidence was also reported for responses made in the absence of remembering, analysis confirming accuracy was significantly higher at the highest bin of confidence ≥ 76 (*M* = 0.71) than for all other bins [(51–75, 26–50 and 0–25, *M* = 0.60, 0.55 and 0.56 respectively, independent two-sided samples *t*-tests all significant *p* = 0.003, *p* < 0.001, *p* < 0.001, (Cohen’s *d* = 0.23, 0.33 and 0.3 respectively)].

**Figure 4:**
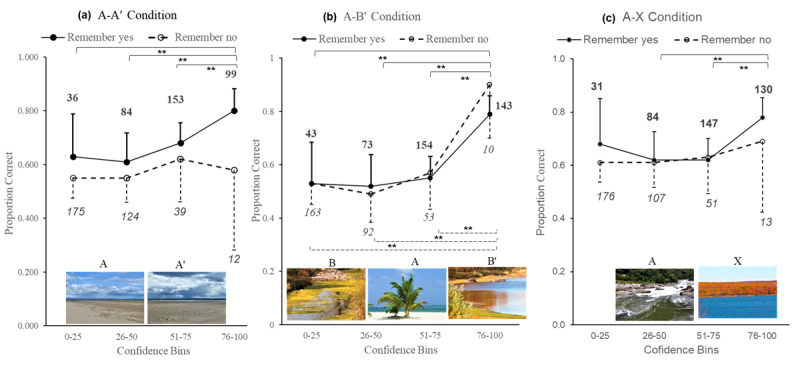
Study 1: CAC plots by test picture-pair condition illustrating recognition accuracy (mean proportion correct) by confidence (binned in quartiles) split by the experience of remembering, **(a)** when lures were perceptually similar (the A–A’ condition), **(b)** when lures were mnemonically similar (the A–B’ condition), **(c)** when lures were dissimilar to any picture shown at study (the A–X condition). Example picture-pairs are illustrated for each condition, all the A target pictures had been shown during the study phase. The B picture was shown during the target block in the study phase of the experiment. Neither A’, B’ nor X images had been shown previously in the study. [(** *p* < 0.001), (* *p* < 0.01). Error bars represent 95% *C.I*. – for clarity only positive or negative bars are shown; numbers represent trials assessed within each bin of confidence – bold figures represent remembered trials and italic figures represent trials associated with not remembering].

We also examined CAC plots by test-pair condition, as shown in Figure 4, unpacking the overall data pattern shown in Figure 3b. Across all test-pair conditions, the proportion of trials where remembering was reported increased as binned confidence was higher. Remembering had the greatest impact on performance when the test pairs were perceptually similar (the A–A’ condition). The overall proportion correct (i.e., accuracy) was significantly higher for the highest bin of confidence ≥ 76; (*M* = 0.80) than for all other bins [(51–75, 26–50, and 0–25, *M* = 0.68, 0.61, and 0.63 respectively). More importantly, when the recognition decision was made in the absence of remembering there was no relationship between recognition accuracy and confidence, i.e., performance on the task was flat, despite higher reported confidence, and with no significant differences in accuracy by bins of confidence (Figure 4a).

CAC plots in the A–A’ condition revealed remembering to be associated with higher recognition accuracy at the highest bin of confidence; the calculations differing significantly at the highest confidence bin ≥ 73, remembering vs. not remembering (*M* = 0.80, 0.58), independent two-sided samples *t*-tests (p = 0.02), Cohen’s *d* = 0.51.

Similarly, in the A–B’ condition (Figure 4b) when remembered, the overall proportion of correct responses was significantly higher at the highest bin of confidence ≥ 76 (*M* = 0.79), than for all other bins, [(51–75, 26–50, and 0–24, *M* = 0.55, 0.52, and 0.53), independent two-sided samples *t*-tests all highly significant *p* < 0.001, (*d* = 0.53, 0.61,and 0.59)]. However, accuracy was also significantly higher in the absence of remembering at the highest bin of confidence ≥ 76 (*M* = 0.90) than for all other bins [(51–75, 26–50 and 0–25, *M* = 0.57, 0.49 and 0.53 respectively, independent two-sided samples *t*-tests all highly statistically significant, *p* < 0.001, (Cohen’s *d* = 0.7, 0.84 and 0.75]. Critically, higher accuracy was associated with the highest bin of confidence whether or not the participant reported the experience of remembering. Noting the discrepancy in number of trials contributing to each data point, the observed difference in accuracy at high confidence ≥ 76 for remembering (*M* = 0.79) compared with not remembering (*M* = 0.9), was not significant (*p* = 0.4).

When test pairs were dissimilar (the A-X condition), performance in the task was essentially flat regardless of remembering, suggesting that confidence was based on factors other than accuracy.

For each condition, we conducted a 4 (confidence bins) x 2 (remembering) ANOVA. Examining data for recognition accuracy (proportion correct) by remembering (no = 0, or yes = 1), revealed a statistically reliable main effect for remembering on recognition accuracy in the A–A’ condition, however, the effect size was less than small (Table 2). There was no significant main effect for the relationship between remembering and accuracy in the A–B’ condition, however, the analysis revealed the relationship between confidence and accuracy was significant (Table 2) with a large effect size. There was no significant interaction between remembering and accuracy in this condition.

**Table 2:** Study 1. Results of 2 × 4 ANOVA by test-pair condition, examining trial level data for recognition accuracy (proportion correct) by remembering (no = 0, or yes = 1), across the four bins of confidence.


STUDY 1		MAIN EFFECT	*F* (*df*)	*p* VALUE	h2	POWER

Condition	A–A’	Remembering	4.56 (1,723)	0.035	0.006	0.56

	A–B’	Confidence bins	5.2 (3,730)	0.001	0.021	0.93

	A–X	none	–	–	–	–


Finally, in the A–X condition there were no significant main effects. The analysis confirmed that only when targets and lures were perceptually similar, the A–A’ condition did remembering the target have a significant main effect on accuracy. In the A–X condition, although remembering was shown to have some effect on accuracy in the CAC plots, the pattern was inconsistent. The ANOVA demonstrated there was no main effect of remembering. Critically, when mnemonically similar lures were presented, the A–B’ condition, higher confidence was seen at higher levels of accuracy regardless of whether the experience of remembering was associated with the recognition decision, with no main effect of remembering.

#### Moderation Analysis

**Figure 5:**
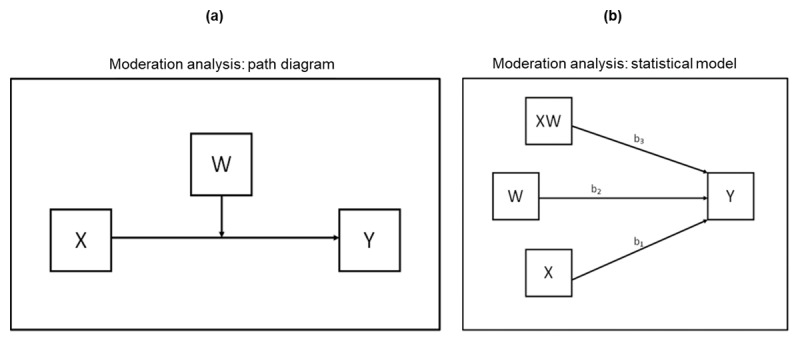
Moderation analysis. **(a)** path diagram: note that the hypothetical variable names (X, Y, W) are changed in the PROCESS macro for SPSS to match the variable names in the analysis (Hayes & Scharkow, 2013; Hayes, Montoya & Rockwood, 2017) where the moderator variable [(W) = remembering or memory vividness], can be dichotomous or continuous for the analysis, the independent predictor variable [(X) = accuracy], can be dichotomous or continuous for the analysis, and the dependent variable [(Y) = confidence], must be continuous for the analysis; **(b)** statistical model: this represents the interaction (XW) tested, see Hayes (2017).

To further scrutinise the three-way relationship, we next asked whether the association between accuracy and confidence was moderated by remembering or its quality (the remembered vividness of the target). While a two-way ANOVA estimates how the mean of a quantitative variable changes based on two categorical variables, it also assesses the main effect of each independent variable and whether there is an interaction between them. By contrast, moderation analysis measures and tests how an independent variable affects the dependent variable, based on a moderator variable that changes the strength of the relationship between the other two variables. We therefore carried out two additional analyses which allow the effect of recognition accuracy on confidence to differ across levels of each moderator (illustrated conceptually in Figure 5).

**Figure 6:**
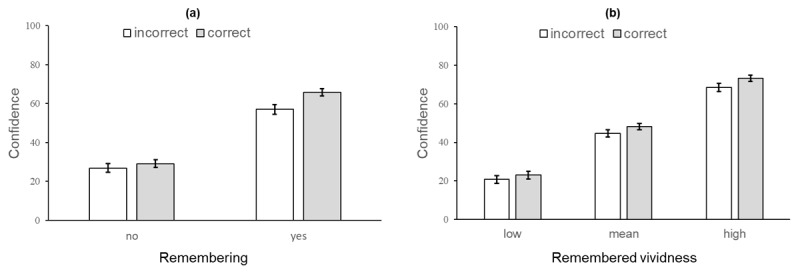
Study 1. Column plots of results from the two moderation analyses (error bars represent 95% C.I.): **(a)** recognition accuracy (incorrect or correct) with confidence by remembering (no or yes); **(b)** recognition accuracy (incorrect or correct) with confidence by vividness at the mean ± 1 S.D., (low, mean, and high = 22, 51, and 80 respectively). The interaction (moderation) effect can be seen by comparing the columns for incorrect versus correct recognition in each case, i.e., representing the three-way interaction (a) between remembering, confidence, and recognition accuracy and (b) between remembered vividness, confidence, and recognition accuracy.

We hypothesised that the strength of the positive effect of accuracy on a participant’s confidence would vary according to whether or not the picture selected as old was remembered, and the quality of the memory, i.e., its remembered vividness. The first analysis revealed that remembering had a significant moderation effect on the relationship between accuracy and confidence, i.e., there was a significant interaction between accuracy and remembering (*p* = 0.002), as shown in Figure 6a. The experience of remembering had a large effect on confidence levels, regardless of recognition accuracy. There was a further small but significant increase in confidence for accurate (correct) recognition compared to incorrect recognition when the experience of remembering informed the decision. Critically, however, in the absence of remembering, accuracy had no significant effect on confidence. The second moderation analysis revealed that remembered vividness had no significant moderation effect on the relationship between accuracy and confidence, with no significant interaction between accuracy and remembered vividness (*p* = 0.09), as shown in Figure 6b. The quality of remembering had a large effect on confidence levels, regardless of recognition accuracy, with no significant difference in confidence associated with remembered vividness for accurate (correct) recognition compared to incorrect recognition at any level. In sum, therefore, the moderation analysis confirmed that the association between recognition accuracy and confidence is moderated by the experience of remembering the target, but not by the quality of remembering (remembered vividness of the selected image).

#### Correlational Analysis

**Figure 7:**
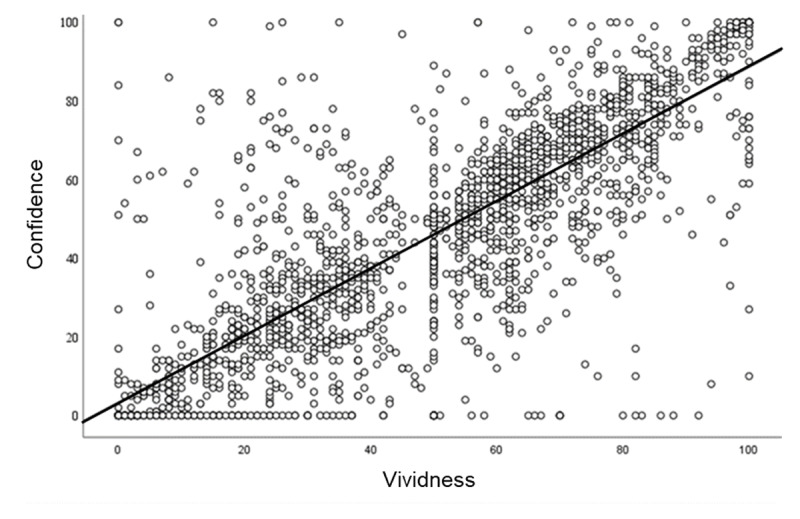
Study 1. Scatterplot of trial level data illustrating the correlation between the vividness of memory for the image recognised as old and confidence in the accuracy of the decision, overlaid is the linear regression (best fit) line.

Because our measures of confidence and vividness were both continuous, we were also able to evaluate the correlation between these variables using trial level data. As can be seen in Figure 7 there was a strong correlation between memory quality (remembered vividness) and confidence in memory’s accuracy (Pearson’s *r* = 0.82, *p* < 0.001). Note that the sliding scale used to make vividness ratings had a starting position of 50, resulting in some anchoring, as seen in Figure 7. We changed the slider starting position to zero halfway through the experiment to encourage participants to move the slider to provide a vividness score. Noting that some anchoring is also visible at 0 for the confidence responses, we point out that participants were instructed to leave the confidence rating at 0 if they were guessing. Despite the presence of anchoring a strong correlation is clearly visible within the data, with two-thirds (67%) of the variance in confidence explained by vividness of memory for the recognised images. The same strong positive correlation was found for data averaged across trials for each participant (aggregated data, *r* = 0.75, *p* < 0.001), and for trial level data in each test-pair condition (A-A′ *r* = 0.8, A-B′ *r* = 0.82, A-X *r* = 0.81, all *p* < 0.001).

Because our measures of accuracy and remembering were both dichotomous, equivalent trial level analyses could not be carried out for these variables. We therefore examined the relationship between these variables and our continuous measure of confidence, using aggregated data for each participant. Analysis revealed a moderate correlation between remembering and confidence (*r* = 0.25; *p* = 0.004), whereas the correlation between accuracy and confidence was not significant (*r* = 0.12; *p* = 0.18).

#### Post-Experiment Questionnaire

Participants were required to complete an online post-experiment questionnaire, as detailed in Supplementary file 1: Appendix. Study 1 Online instructions to participants, producing self-report data about what decisions were based on. These data allowed us to confirm that participants had followed the instructions regarding the remember response, with the majority of remember responses being associated with conscious awareness relating to a personal memory. Bearing in mind that participants were instructed to check all responses that applied, the commonest reason cited for remembering the target was that it reminded the participant of something (69% of all responses). The second most common response was that the participant remembered thinking of an association or a personal memory (53%), followed by remembering what they were thinking when first viewing the image (47%). Liking the picture or thinking it was attractive was also common (42%, representing a positive emotional response), whereas only a few responses were associated with remembering something happening in the room at the time of viewing the picture (9%). In the absence of remembering, the commonest reason cited was not recognising the other picture in the test-pair (64%), with little difference between random guessing (48%) or thinking the picture was familiar (52%). When participants were not forced to answer remember or know, the reason for the decision that was reported least often was just ‘knowing the picture was old’ (30%).

### Discussion

We asked whether confidence in the accuracy of recognition is affected more by the experience of remembering or by its factual accuracy. Across conditions, our data show that remembering had the stronger effect on confidence. Conversely, CAC plots for the overall data confirmed highly accurate recognition was associated with high confidence (corresponding with reports in the wider literature, e.g., Brewer & Wells, 2006), with no significant effect of remembering. Critically, however, by examining performance across the three test-pair conditions, our data reveal that the relationship between confidence and accuracy varies as a function of the context in which to-be-remembered stimuli are presented at test. CAC plots revealed that links between high accuracy and high confidence for remember responses were present when lures were perceptually or mnemonically similar. Analysis confirmed that although remembering only had a significant effect on accuracy in the perceptually similar A–A’ condition, in the mnemonically similar A–B’ condition confidence was also higher for highly accurate responses, but this was not reliant on the decision being made with the experience of remembering. Analysis also revealed that remembering had no significant effect on accuracy in the A–X condition (i.e., when lures were unrelated to targets). Apart from when mnemonically similar (A–B’) lures were presented at test, there was no relationship between accuracy and confidence in the absence of remembering.

Our findings replicate Tulving’s confidence-accuracy inversion of lower mean confidence and higher mean accuracy for perceptually similar (A–A’) pairs compared to higher mean confidence and lower mean accuracy for mnemonically similar (A–B’) pairs. Nonetheless, on the basis of the different CAC plot patterns seen across conditions, our data suggest a novel explanation of the confidence-accuracy inversion. For A–A’ pairs higher confidence was seen for remembered stimuli across all bins, with highest levels of confidence predominantly seen with the experience of remembering and with high accuracy. For mnemonically similar (A–B’) pairs, the highest bin of confidence linked to high accuracy regardless of remembering. We suggest, therefore, that in some cases high confidence can occur due to participants thinking they have remembered the target while mistakenly remembering a mnemonically similar lure (i.e., misremembering as old a B’ lure that is similar to a B target shown at study). Higher levels of confidence linked to inaccurate ‘remembered’ responses may be responsible for the inversion effect of lower accuracy and higher confidence compared to A–A’ pairs. This view is supported by wider evidence that overlap between features of stored information may explain autobiographical memory errors (Gonsalves & Paller, 2002; Newman & Lindsay, 2009).

Additionally, and reflecting the accessibility model of the feeling of knowing, (Koriat, 1993), participants retrieve information from memory as the basis for their confidence ratings, using whatever they remember from the search process (regardless of accuracy) to inform their recognition decision. We also comment that we found mean accuracy to be highest in the A–X condition. When the lure was dissimilar to the target or to any other picture studied, neither remembering nor confidence was reliably associated with changes in accuracy, with self-report data suggesting that not recognising the lure could be relied on. The present results suggest, therefore, that a new stimulus can trigger recognition, e.g., if the stimulus elicits a personal memory. Note that similar effects are also likely to occur in eyewitness memory, for example, if an innocent party’s face in an eyewitness lineup looks similar to the face of a neighbour or a former acquaintance, meaning that the eyewitness believes they accurately recognised the face, particularly if detail, i.e., the source of the memory, is lacking.

Taken together, therefore, our data indicate that confidence in the accuracy of recognition is based largely on the experience of remembering, rather than on knowledge of its accuracy. Moderation analyses confirmed that the association between recognition accuracy and confidence was differentially affected by the experience of remembering. In addition, despite the presence of a strong correlation between memory vividness and confidence, memory vividness did not significantly moderate the association between recognition accuracy and confidence. The lack of a differential effect may, however, reflect little more than the fact that vivid images are recognised more accurately (i.e., due to the paucity of the dichotomous judgment required in old or new recognition tasks). Consequently, to triangulate our findings, we carried out a second study using a source memory task that provides a continuous measure of accuracy.

## Study 2 – Source Memory Accuracy, Memory Vividness and Confidence

### Introduction

Source memory paradigms are based on recollection (remembering) and consist of two pieces of information, the first representing recognition memory for an item and the second representing recollection of the context in which the information was presented. The latter, referred to as source memory, is considered an essential part of episodic memory (cf. Johnson, 2005; Johnson, Hashtroudi & Lindsay, 1993). From the extant literature we theorise that memory quality (vividness) will influence confidence that memory for an event is accurate. For example, in an eyewitness task, Robinson et al., (2000) confirmed a disconnect in confidence between accurate and inaccurate recognition, concluding that metamemory monitoring was closely related to the vividness of the recollected information, and pointing to vividness being an important mediator of recognition-recall differences.

Recollection (recall) of contextual details experienced at the time of encoding, i.e., the who, what, and when of memory, is rarely assessed in a typical old or new recognition memory experiment (Mandler, 1980). This is of relevance for those working in the eyewitness field, where in court, the eyewitness may well be asked not only about the who, but also about the what, and when of their memory. By contrast evidence linking memory vividness and confidence is particularly clear in studies of source memory, providing a strong rationale for examining these relationships using tasks that require recollection of contextual detail (e.g., Cuperus, Laken, van Schie, Engelharde & van den Houte, 2019; Leshikar & Duarte, 2012; Richter et al., 2016).

Following this approach, Study 2 employed a novel source memory paradigm (Harlow & Donaldson, 2013; Harlow & Yonelinas, 2016; Murray, Howie & Donaldson, 2015), initially devised to test theoretical accounts of the nature of recollection. Importantly, the task provides a continuous measure of memory accuracy, based on the participant’s ability to recollect information about the location of a cross on a circle (cued by a word associated with that location at study). To assess memory vividness the task was adapted, using real-world natural scene images (rather than words) as retrieval cues. Study 2 therefore delivers a unique combination of continuous data for source memory accuracy, memory quality (vividness), and confidence, allowing us to re-evaluate the findings of Study 1. In the continuous source memory paradigm, recollection of the cross location only occurs if the associated picture has been remembered (see Harlow & Donaldson, 2013). That is, if participants do not remember the picture, they cannot recollect the cross location and have to respond by guessing. The source memory task therefore provides a continuous measure of the accuracy of recollection and participants do not need to be asked whether or not they had the experience of either recognising the image or recollecting the cross location.

Two experiments were carried out in Study 2. We initially used 120 images from the same database of natural scene photographs in full colour, as described for Study 1, allowing the creation of 120 unique natural-scene plus cross-location stimulus pairs for presentation. However, one criticism that could be made of Study 2, Experiment 1, is that because all the images were in full colour they may have been equally vivid at study and at test (in terms of their physical properties, i.e., external vividness – sharp contrast, different colours), which might have forced participants to give subjective ratings of vividness on other dimensions. To counter this concern we repeated the study for Experiment 2, where the 120 images in full colour were augmented by the addition of 120 different natural scene photographs, partially desaturated for colour and not adjusted for contrast.

### Methods

#### Recruitment

A total sample size of 32 participants provides sufficient statistical power (0.8) to assess a between-groups correlation (Pearson’s *r*) at 5% significance level (two-tailed). Participants comprised undergraduate and postgraduate students from the University of Stirling, naïve to the aim of the study, with inclusion criteria as for Study 1, and approved by the University of Stirling’s GUEP. All participants gave written informed consent and volunteered their time; psychology undergraduates took part for mandatory course credits.

### Study 2 – Experiment 1

#### Stimuli

**Figure 8:**
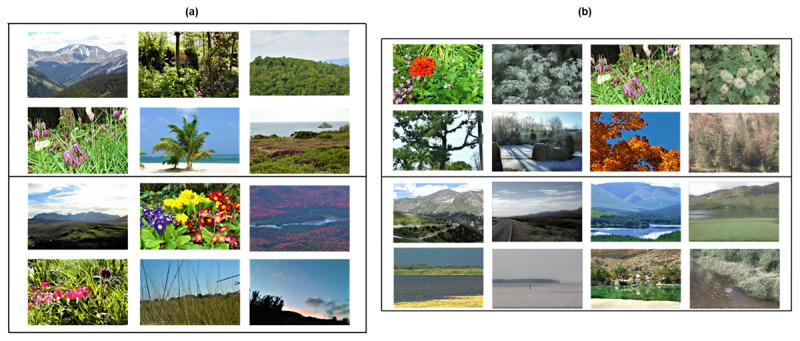
Study 2. Example natural scene colour photographs used for both study and test phases, comprising pictures from each subject category of flowers, green landscapes and trees, mountains, and water, used for: **(a)** Experiment 1, in full colour – images were counterbalanced for subject category; **(b)** Experiment 2, half in colour and half partially desaturated for colour – images were counterbalanced for subject category and colour saturation.

In Experiment 1 stimuli consisted of 120 unique natural-scene photographs each paired with a unique cross-location. These pairs were presented during relational encoding trials (split into 10 blocks of 12) as outlined below. Blocks were counterbalanced for subject category, with each block comprising a random selection of three different images of flowers, green landscapes and trees, mountains, and water (as illustrated in Figure 8a). In Experiment 2, 240 unique natural-scene plus cross-location stimulus pairs were presented during relational encoding trials (split into 15 blocks of 16). Blocks were counterbalanced for subject category and colour saturation, each block comprising a random selection of four different images of flowers, green landscapes and trees, mountains, and water (see Figure 8b). Experiment 2 allowed Experiment 1 to be reproduced using different participants and image sets with manipulation of the external vividness of half of the photographs.

#### Procedures

Experiments were conducted in person (with the researcher present) in a designated quiet psychology testing room using E-Prime (version 2.0 Professional, Psychology Software Tools Inc.), on a laptop PC, with photographs presented in landscape orientation on a white background, at a resolution of 640 × 425 pixels. Experiments involved a single session of 30–40 minutes for each participant, all carrying out a short practice using eight different picture-location pairs before commencement.

##### Relational encoding (study) phase

**Figure 9:**
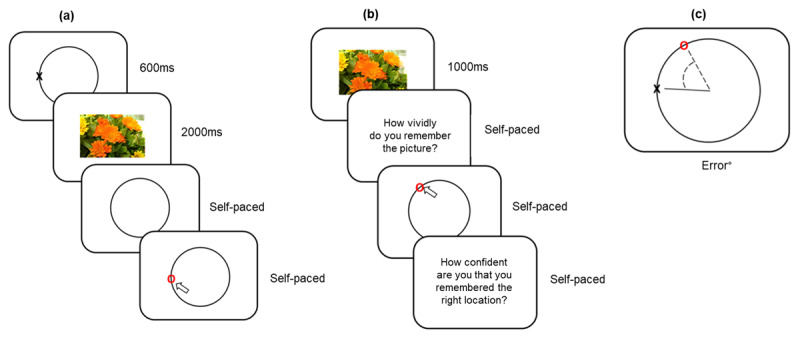
Study 2. Protocol for Experiments 1 & 2: **(a)** Study phase. Relational encoding is required during trials – photographs are presented following a location shown as a cross on a circle; **(b)** Test phase. Photographs are re-presented, and the location of the associated cross must be recollected, judgments of vividness, accuracy and confidence are required during trials; **(c)** Source memory accuracy, measured based on radial error: the distance in ± degrees between the participant’s response (shown as a red circle) and the correct target location (shown as a cross on the circle).

Encoding trials involved the presentation of a series of images plus an associated target (cross) location, shown sequentially within relational encoding blocks (12 trials per block in study 1; 16 in study 2). As shown in Figure 9a, at study trials commenced with the presentation of a black cross located on a grey circle (600 ms), followed by a natural scene photograph (2000 ms). Participants were explicitly told to pay attention both to the cross location and the related picture to be able to perform the task. Participants verified their attention by indicating the (now hidden) cross location using the mouse (self-paced). Responses within 6° of the cross location advanced participants to the next trial, otherwise the cross location was re-presented (250 ms) and the verification task repeated as described. The task can be completed with accuracy, i.e., the binding of the cross location and the retrieval cue is not affected by their consecutive presentation (Harlow & Donaldson, 2013; Murray et al., 2015).

##### Retrieval (test) phase

Each relational encoding study block cross location picture-pairs was immediately followed by its corresponding test phase. Introductory instruction screens between study and test ensured that participants could not rely on working memory. Trial phase and photograph presentation order was randomised by E-prime during both study and test phases (to counterbalance any effect of list order). During retrieval, pparticipants were shown each of the previously presented photographs (1000 ms) from the corresponding relational encoding phase, i.e., all test images were old, as shown in Figure 9b. Following each photograph, the participant was presented with a circle and was asked to indicate the related cross location using the mouse (self-paced), requiring recollection of information associated with the cue picture. Radial error data (0° to ±180°) for each trial was based on the distance in ± degrees between the participant’s response and the correct target location, as illustrated in Figure 9c. A continuous measure of source accuracy for each individual trial was provided by expressing the possible error as a percentage (such that 0° error = 100% source accuracy, ± 90° error = 50% source accuracy, ± 180° error = 0% source accuracy). If participants could not or did not perform the task and were unable to recollect the locations, i.e., were guessing throughout the experiment, the location error frequency plot would be flat (signifying no recollection), allowing the quality of the final data sets to be monitored and their data excluded. (cf. Zhou, Osth, Lilburn & Smith, 2021).

##### Behavioural data

Behavioural data were assessed during the test phase (Figure 9b). Because only old stimuli were presented no recognition decision was required. In response to each test image participants were asked to make an initial vividness judgment (‘how vividly do you remember the picture?’) using a 100-point scale. Next participants were asked to recollect the location associated with the image by moving a mouse to the correct position on a circle. Finally, participants were asked to make a confidence judgement (‘How confident are you that you remembered the right location?’), again using a 100-point scale. Full details of the instructions and response requirements are provided in Supplementary file 3: Appendix. Study 2, Experiments 1 & 2, instructions to participants.

#### Data Analysis

Data were analysed according to the methods reported for Study 1.

### Results

#### Participants

Experiment 1 involved 16 participants, mean age 21 years (*S.D*. 2 years); 14 identified as female; data from all participants (1920 trials) were included in the final analysis. Experiment 2 involved 16 participants, mean age 20 years (*S.D*. 3 years); 15 identified as female; data from 13 participants (3120 trials) were included in the final analysis; data were excluded from three participants whose location error frequency plots were flat, indicating guessing. The final data set comprised 5,040 trials across 29 participants in both experiments [power = 0.77 for tests of correlation between independent groups; power for *t*-test comparison (two-tailed) = 0.25 (critical *t* = 2.05, *df* = 27; *p* ≤ 0.05)].

#### Variables of Interest

Variables of interest were compared by experiment using trial level data. Remembered vividness, confidence, and source memory accuracy were all significantly higher in Experiment 1 (cf. Table 3). Effect sizes were weak for accuracy, and weak to moderate for vividness and confidence.

**Table 3:** Study 2. Comparison of variables of interest: data are compared by experiment and assessed using unstandardized B coefficient and R^2^ change (*N* = number of trials).


EXPERIMENT (*N*)	VARIABLE	*M* (*SE*)	*F* (*df*)	UNSTANDARDIZED B COEFFICIENT (*SE*)	*p*-VALUE	*R^2^* CHANGE

Experiment 1 (1920) Experiment 2 (3120)	Vividness	67.4 (0.6) 55.3 (0.4)	187.1 (1,5038)	–12.31(0.86)	< 0.001	3.9%

Experiment 1 (1920) Experiment 2 (3120)	Confidence	54.9 (0.7) 42.6 (0.6)	205.5 (1,5038)	–12.3 (0.90)	< 0.001	3.6%

Experiment 1 (1920) Experiment 2 (3120)	Accuracy	74.1 (0.6) 67.6 (0.6)	56.67 (1,5038)	–6.55 (0.87)	< 0.001	1.1%


**Figure 10:**
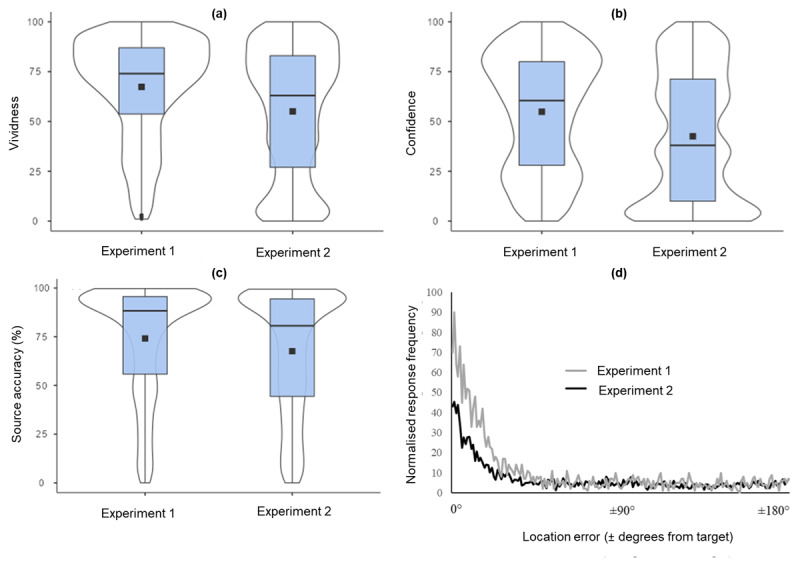
Study 2, Experiments 1 & 2, box plots illustrating median values (horizontal black bands) and the associated violin plots of responses illustrating mean values (black squares) reflecting the effect of changing the visual vividness of the pictures on **(a)** remembered vividness, median = 74 and 63, respectively; **(b)** the distribution of confidence in the memory’s accuracy, median 60.5 and 38, respectively; **(c)** source accuracy, median = 88.3 and 80.6, respectively; (d) location error response frequency plots showing source memory performance for each degree of error from the target (0° to ± 180°, normalised by number of trials and number of participants).

Box plots and overlaid violin plots illustrate the distribution of remembered vividness (Figure 10a), confidence ratings (Figure 10b) and source accuracy (Figure 10c). Notably, the distribution of source accuracy responses was similar but not identical across experiments, reflecting more trials of lower accuracy and fewer trials of high accuracy in Experiment 2, due to the addition of pictures manipulated to be less visually vivid. The difference in source accuracy between experiments is further illustrated by the plot of source memory errors (Figure 10d, modelled using methods introduced by Harlow & Donaldson, 2013). Results confirm that source accuracy was reduced in Experiment 2, i.e., fewer responses were seen close to 0° (the target location).

#### Confidence Accuracy Characteristic (CAC) analysis

**Figure 11:**
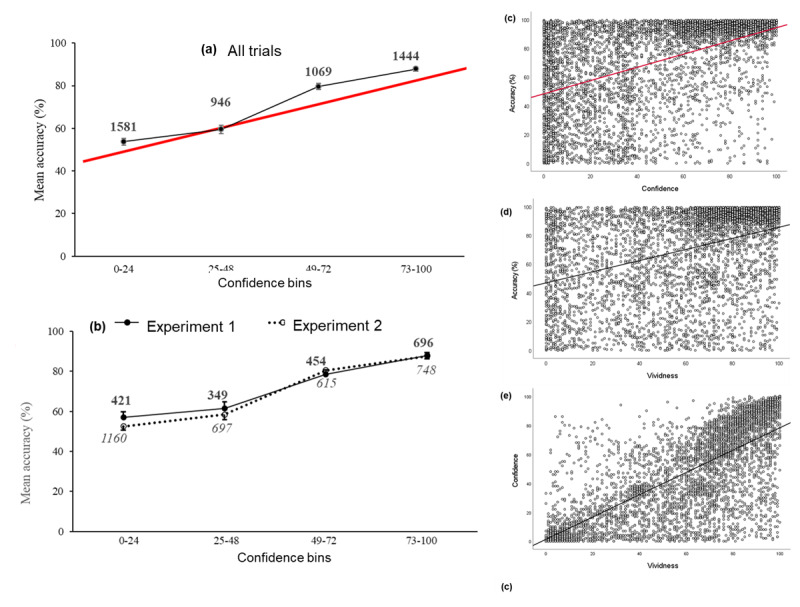
Study 2. **(a)** combined trial level data from Experiments 1 & 2 illustrating the association between source accuracy and confidence as a calibration (CAC) plot of percentage accuracy averaged for each bin of confidence. The red line superimposed on the plot reproduces the linear regression line from the scatterplot (c) of confidence and accuracy; **(b)** trial level data from Experiments 1 & 2 illustrating the association between source accuracy and confidence by experiment as a calibration (CAC) plot of percentage accuracy averaged for each bin of confidence. (Error bars represent 95% *C.I*., numbers represent total trials for each bin of confidence); **(c)** combined trial level data from Experiments 1 & 2 illustrating the association between confidence and source accuracy as a scatterplot; **(d)** combined trial level data from Experiments 1 & 2 illustrating the association between vividness and source accuracy as a scatterplot; **(e)** combined trial level data from Experiments 1 & 2 illustrating the association between vividness and confidence as a scatterplot. (The best fit linear regression lines are superimposed).

CAC analysis was used, as for Study 1, to explore the relationship between source memory accuracy and confidence for all trials across the combined experiments (capturing a full range of remembered vividness). Confidence (mean, *M* = 47.3, median = 48) was binned into quartiles of 24, 48, and 72, with average accuracy calculated for each bin, as shown in Figure 11a. The plot clearly illustrates that as accuracy increases higher confidence is reported. Accuracy was significantly higher for confidence ≥ 73 (*M* = 87.8) compared to all other bins (49–72, 25–48, and 0–24; *M* = 79.6, 59.5, and 53.7 respectively), with all independent two-sided samples *t*-tests, *p* < 0.001 (Cohen’s *d* = 0.39, 1.16, and 1.35 respectively). Comparing Experiments 1 and 2, the pattern of source memory accuracy across binned confidence mirrored that of the combined data, as can be seen in Figure 11b, and justifying our use of the combined data set.

#### Correlation analysis

In comparison to Study 1, the continuous nature of source accuracy data enabled by trial correlation analysis to be carried out to further assess the strength of the relationship between accuracy and confidence for the combined experiments. We next examined the association between source memory accuracy and confidence using both a scatterplot (Figure 11c) and a calibration plot (Figure 11a). Counterintuitively, the scatterplot has confidence on the x-axis. Being aware that accuracy is not dependent on confidence, the adjustment is made to match the calibration plot in Figure 11a. Correlational analysis revealed a moderate correlation (*r* = .48) between source accuracy and confidence for the combined experiments, just over 20% of the variance in confidence could be explained by variability in source memory accuracy (see Table 4 for results by experiment).

**Table 4:** Study 2. Correlations between variables of interest (across all trials) within each Experiment. Data are compared using Pearson’s correlation coefficient (*r*) and variance (R^2^).


EXPERIMENT	I.V.	D.V.	*N*	PEARSON’S COEFFICIENT (r)	*p*-VALUE 2-TAILED	*R^2^* VARIANCE

Experiment 1	Accuracy	Confidence	1920	0.46	< 0.001	20.8%

Experiment 2	Accuracy	Confidence	3120	0.48	< 0.001	23.3%

Experiment 1	Vividness	Confidence	1920	0.65	< 0.001	**42.3%**

Experiment 2	Vividness	Confidence	3120	0.76	< 0.001	**57.3%**

Experiment 1	Vividness	Accuracy	1920	0.33	< 0.001	10.7%

Experiment 2	Vividness	Accuracy	3120	0.39	< 0.001	15.9%


Notably, when superimposed on the CAC plot the best fit linear regression line from the scatterplot of all trial data (Figure 11c) reproduces the line of the calibration plot (Figure 11a). By comparison, the scatterplot provides far richer information about the data distribution compared to the calibration plot. For example, when low confidence responses (lowest quartile) are examined, accuracy appears to be at close to chance levels (53.7%), suggesting that performance largely reflects guessing rather than veridical remembering. More importantly, when high confidence responses (highest quartile) are examined accuracy is far higher (87.8%), but there are still substantial numbers of trials for which accuracy is low. That is, guessing still contributes considerably to performance when confidence is high, such that confidence cannot reliably be used to infer the accuracy of remembering.

Although our by-trial correlation analysis reveals a clear outcome, (see Table 3 for correlations by experiment) analytical methods can produce differences in correlation results (Roediger & DeSoto, 2014), particularly when relationships differ between individuals. We therefore repeated the analysis using averaged data across participants. Correlation of within-participant averaged data showed the association between accuracy and confidence to be strong (*r* = 0.78), as expected, given wider evidence that correlations can be stronger when averaged data are assessed (see Aggarwal & Ranganathan, 2016). The average of individual correlations between accuracy and confidence gave a weaker association, overall (*r* = 0.42), and for experiments 1 and 2 (*r* = 0.42, 0.43, respectively), all correlations being positive.

Table 3 compares results of by-trial data correlations for each experiment. Reproducing results from Study 1, the relationship between remembered vividness and confidence for the combined experiments was strong (*r* = 0.74; Figure 11d); around 50% of the variance in confidence could be explained by variability in the remembered vividness of the cue picture with an obvious linear relationship. In comparison to the scatterplot in Study 1 (cf. Figure 8), more trials were seen in the bottom right-hand corner of the plot, i.e., higher remembered vividness (> 50) of the cue image could be associated with lower confidence (< 50) for source memory recollection compared to confidence in recognising the image. In contrast, the relationship between vividness and source memory accuracy for the combined experiments was weak (*r* = 0.39; Figure 11e). Although trials with higher remembered vividness of the cue image were more likely to be associated with higher source accuracy (recollection of associated contextual detail), the relationship between vividness and accuracy is less evident than that between vividness and confidence.

#### Moderation Analysis

**Figure 12:**
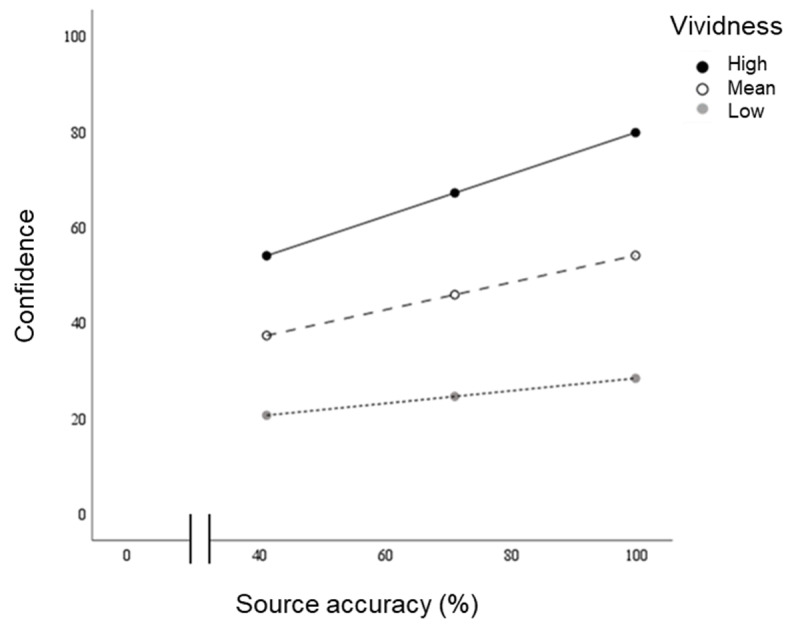
Study 2. Experiments 1 & 2 combined: results of the moderation analysis illustrated by simple slopes plots (of the interaction effect) showing the relationship between source memory accuracy and confidence, by vividness at the mean ± 1 S.D., (high, mean, and low = 89.9, 59.7, and 29.5 respectively).

Analysis thus far suggests that memory quality (i.e., remembered vividness) has a stronger association with confidence than with accuracy. We hypothesised, however, that the strength of the positive effect of source accuracy on a participant’s confidence would vary according to the vividness of memory for the associated cue image. Put simply, the vividness of memory should moderate the relationship between accuracy and confidence (see model illustration in Figure 5). Analysis revealed that the interaction between accuracy and vividness on confidence was significant (*p <* 0.001), with a weak effect size (*R^2^* change = 1.9%). This result is illustrated in Figure 12 using a simple slopes plot, showing the conditional effects of accuracy on confidence at different values of the moderator. At low, mean, and high levels of vividness (29.5, 59.7 and 89.9 respectively), the conditional effect of accuracy on confidence is represented by the slope [*B* = 0.13, 0.27 and 0.42 respectively; all *p* values < 0.001]. Critically, the slope becomes steeper with increasing memory vividness. The association between source accuracy and confidence was therefore conditional on the remembered vividness of the cue picture, i.e., for the same level of accuracy, confidence was higher, with the effect also increasing (the curves separate) as accuracy increased.

#### Subsidiary Experiments: Judgments of Vividness at Study

Our perception of vividness may differ when experiencing compared to when remembering a stimulus (e.g., with images judged as less “visually salient” when remembered than when experienced, cf. Cooper et al., 2019). To address the potential concern that participants may not have been reporting the quality of their memory for the picture at test, but instead simply reporting how vivid the picture seemed to them when viewed again, we repeated the Study 2 experiments with vividness judgments made during the relational encoding phase, rather than at test see Supplementary file 4: Appendix. Study 2, Subsidiary experiments judging vividness at study, concluding that participants in Experiments 1 and 2 were assessing the vividness of their memory for the pictures.

### Discussion

Using a continuous source memory task in Study 2 and replicated across two experiments that evoked varying degrees of visual vividness, we again demonstrated a strong association between confidence in source memory accuracy and the quality of remembering (the remembered vividness of the cue photographs). Consistent with previous claims, CAC plots confirmed that higher source accuracy was associated with confidence above the median. In contrast to Study 1, however, the findings of Study 2 highlight that examining memory data using scatterplots provides greater resolution than is available in CAC plots. By their nature, scatterplots reveal variability at the level of individual trials that is hidden within averaged data. In the present case, examining this variability shows that although confidence correlated with accuracy overall, guessing contributed significantly to performance even when confidence was high (cf. Figure 12a). Crucially, therefore, in any single instance of remembering confidence can be high even though memory is inaccurate. In addition, our analysis also demonstrated that remembered vividness had a significant moderation effect on the relationship between accuracy and confidence, confirming that confidence (rather than source memory accuracy) was differentially affected by the quality of remembering. These results are consistent with reports from the literature (as discussed in the introduction) showing that contextual details of vivid memories may not always be accurate. The findings from Study 2 therefore provide further support for the claim that confidence in memory is related more to remembering and its quality, than to its factual accuracy.

Study 2 also highlights the importance of decisions about the level at which the relationship between accuracy and confidence is examined. We found the correlation between accuracy and confidence to be moderate in Study 2, despite very large correlations being reported previously using old or new recognition tasks. We take the view that the difference in the strength of the correlation most likely reflects a difference in the level at which the data was analysed (e.g., Roediger & DeSoto, 2014). In studies that rely on dichotomous responses (e.g., old or new recognition or binary source judgments) data is primarily analysed using participant-level averages. By contrast, our use of a task that provides a continuous measure of source memory accuracy allowed data to be analysed at the level of individual trials. Here, therefore, results illustrate that the correlation between accuracy and confidence is exaggerated when averaged participant-level data is examined.

## General Discussion

Across two separate studies we demonstrated that confidence in recognition accuracy and confidence in source memory accuracy were both strongly linked to the experience of remembering or its quality (represented by the remembered vividness of natural scene photographs). Study 1 provided definitive evidence that remembering had a stronger effect on confidence than did recognition accuracy, confirmed by moderation analysis. Put simply, the experience of remembering and its quality act as stronger cues than accuracy to inform metacognitive judgments of confidence, although an individual may not be consciously aware this is the case (being certain their recollection is factual even when it is not). Critically, in Study 1, there was no overall relationship between accuracy and confidence in the absence of remembering. We provided further confirmation of these findings in Study 2, using a completely different task, and a continuous measure of memory accuracy. In this case we were also able to examine the correlations between accuracy, vividness and confidence at a single-trial level. The presence of strong correlation between vividness and confidence in both studies leads us to conclude that remembering and its quality drive confidence more than they drive accuracy, providing one explanation for why dissociations between accuracy and confidence may occur.

One notable feature of our data is the fact that remembered vividness moderated the relationship between accuracy and confidence in Study 2, but this was not the case in Study 1. This difference in the effect of remembered vividness might be considered surprising given that we employed the same images in both studies. The lack of a differential effect of vividness on the relationship between recognition accuracy and confidence is unlikely to reflect the change to fully continuous measures in Study 2, however, because we also used continuous measures of confidence and vividness in Study 1. By contrast, the switch from a task that assessed recognition to a task that assessed source memory retrieval may have contributed to the differential effects of vividness. In Study 1 we assessed vividness of memory for the recognised picture and the accuracy of that recognition, whereas in Study 2 we assessed vividness of memory for the cue picture that was associated with memory for the cross location (such that vividness-related increases in confidence for the cue need not necessarily have been related to increases in the accuracy of the associated source memory). In both studies, remembered vividness was strongly associated with confidence. Our findings suggest, therefore, that stimuli that are experienced as vivid may be more likely to be recognised accurately, but although remembered vividness may increase confidence in source memory, it does not guarantee accurate recall of contextual detail.

One important implication of the present findings is in contributing to debate within the eye-witness testimony literature regarding how memory should best be assessed. First, and foremost, our data suggest that confidence judgments should not be used as a proxy for accuracy in eye-witness settings. Even the most confident instances of remembering can be inaccurate. Second, our data reveal that the relationship between confidence and accuracy varies as a function of the context in which to-be-remembered stimuli are presented at test, confirming that the nature of the fillers (or lures) used in line-ups or show-ups is likely to have a substantial impact on performance. Overall, therefore, our results support the views of Colloff & Wixted (2020) in arguing for the use of perceptually similar fillers (i.e., as in our A-A’ condition) because this leads to higher levels of recognition accuracy (cf. Table 1). Nonetheless, our data also provide a crucial proviso for this situation, namely that assessing remembering is vitally important. As shown in Study 1, recognition accuracy is far higher for remembered stimuli, regardless of how confident participants are in their memory (cf. Figure 3a).

We also note, however, that in practice eyewitnesses are considered likely to be less accurate (and more prone to false recognition) if the suspect and fillers are similar. That is, the more similar lures are to targets, or innocent people are to suspects in an identification line-up, the more likely it is that confident false recognition will occur (DeSoto & Roediger, 2014; Grabman et al., 2019; Roediger & DeSoto, 2014). In our task, it is clear that the experience of remembering a picture that was mnemonically similar to a lure led to lower accuracy and higher confidence (i.e., producing Tulving’s inversion effect). Similarly, if an eye-witness’s recognition decision is based on memory for a person who is mnemonically similar to the suspect, this may lead to a highly confident but inaccurate identification. In short, therefore, although our data suggests that assessing remembering is beneficial, it is also the case that assessments based solely on the experience of remembering can lead to decision errors (see Leippe & Eisenstadt, 2007). Indeed, our view is that highly confident false recognition is always possible, regardless of the nature of the composition of the line up (even if the lures are completely unlike, or unrelated to, the target).

## Conclusions

Our results counsel against interpreting high confidence as reliably indicating memory accuracy, even when associated with the experience of remembering or its vividness. Our findings have implications for accounts of vividness, confidence, episodic memory, and eyewitness testimony and offer a novel explanation for the confidence and accuracy inversion in the picture similarity task. High confidence remembering may not in all cases denote high recognition accuracy. Highly vivid memories, confidently recollected, may not always denote the factual accuracy of memory. As a take-home message, we paraphrase Loftus and Greenspan (2017) when they said, ‘If I’m Certain, Is It True?’ Here we urge theorists, practitioners and anyone reflecting on a memory, to ask, ‘If My Memory is Vivid, I’m Certain, But Is It True?’

## Data Accessibility Statement

Our data has been accepted and archived in DataSTORRE: Stirling Online Repository for Research Data and it has been assigned the following identifiers:

**Study 1**

http://hdl.handle.net/11667/238

**Study 2**

http://hdl.handle.net/11667/167

Additionally all images used in Study 1 are available online in DataSTORRE: Stirling Online Repository for Research Data and the file has been assigned the following identifier: http://hdl.handle.net/11667/255.

## Additional Files

The additional files for this article can be found as follows:

10.5334/joc.477.s1 Supplementary File 1. Appendix. Study 1, Recruitment and online instructions to participants.

10.5334/joc.477.s2 Supplementary File 2. Appendix. Study 1, picture similarity criteria.

10.5334/joc.477.s3 Supplementary File 3. Appendix. Study 2, Instructions to Participants.

10.5334/joc.477.s4 Supplementary File 4. Appendix. Study 2, Subsidiary experiments, judging vividness at study.
