# Supplementary material for: Does the Experience of Remembering Differentially Influence the Factual Accuracy of Recognition, and Confidence in Its Accuracy?
Source: J Cogn. 2026 Jan 7;9(1):6. doi: 10.5334/joc.477 (PMC12785665; doi:10.5334/joc.477)
Supplement: Supplementary File 1. — Appendix. Study 1, Recruitment and online instructions to participants. [file joc-9-1-477-s1.pdf]

Supplementary file 1: Appendix. Study 1, Recruitment and online instructions to participants

Recruitment and Data Protection

Due to issues with Covid-19 lockdown restrictions experiments were carried out online using the Gorilla^{TM} cloud-based psychology software testing platform (https://app/gorilla.sc). Recruitment was carried out via Stirling University's Psychology Sign-Up System. To ensure data quality, potential participants were asked to view a short PowerPoint presentation to explain the task and were provided with a link to an approved information sheet before confirming enrolment. To ensure confidentiality and data security, the software provided participants with a unique non-identifiable Private ID (a random anonymous digit string). IP addresses were not collected. Participants were individually invited to log in to the experiment site on a specified date using their personal link, when the study information was repeated, a short demographic questionnaire asked for age and gender identity and electronic informed consent was obtained before the study procedure could begin. In compliance with BPS (British Psychological Society) requirements, identifying data, demographic information and performance data were all stored separately. They were downloaded separately and joined together outside the testing platform using the Private IDs. By default, data from each participant only became accessible when the participant had completed the whole experiment within the allocated time (2 hours).

On-line Instructions

The following on-screen instructions were given to participants prior to starting the test sequence:

"You will see two images together. You will have 6 seconds to decide which picture you have seen before in the study phase. The buttons are labelled left and right, Press the button under the image you think is old. Once you press the button you cannot change your mind and the screen will advance to the vividness rating. A timer will count down the last 3 seconds. Try to remember the image selected for the following ratings. Press Continue when you are ready to start the testing phase." (

Behavioural Data

Vividness.

Participants rated the vividness of their memory for the photograph selected as old (when it was no longer visible on the screen) on a continuous 0 - 100 sliding scale, being instructed:

'Move the slider to rate the picture that you selected as old, for vividness, by answering the question "How vividly did you remember the picture?" There is no time limit. Press continue when you are happy with the slider position.'

(The slider starting position was set at 50; halfway through the experiment the vividness slider starting position was changed to 0 due to observed anchoring.)

Confidence.

Participants rated confidence in their Old or New decision on a continuous 0 - 100 sliding scale, being instructed:

'"Move the slider to rate your confidence in your Old or New decision by answering the question "How confident are you that you remembered the old picture correctly?" Note if you were guessing, your confidence rating should be zero. There is no time limit. Press continue when you are happy with the slider position"'

(The slider starting position was set at 0 for the duration of the experiment.)

Remembering.

Instructions for the remember response were broadly in line with those used by Rajaram, (1993). Participants were given the following onscreen instructions:

> ‘You need to interrogate your memory to answer the Remember question. Choose Remember yes, Only if: you have an experience of recollection for the picture you selected; you are consciously aware of some aspect of what was experienced at the time the picture was presented in the study phase; you have a sense of yourself in the past and/or the picture brings back to mind a particular association, image, or thought you experienced at the time the picture was presented in the study phase. If there was another reason for your decision, just answer no. Think about the instructions before you answer. Once you have pressed the button Remember yes or Remember no, you cannot change your mind and the screen will advance to the next test pair.”

Post-Test Questionnaire

As a quality check on how participants had interpreted the instructions, before leaving the experiment they were asked to complete a short online checkbox questionnaire based on how well they complied with the instructions:

Remembering = Yes.

‘When you answered Yes to remembering the picture, on what did you base your judgment (check all that apply.)’

1) I thought of a personal memory or association when I first saw the picture.
2) The picture reminded me of something.
3) I just thought the picture was attractive - I liked it!
4) I remembered that something happened in the room when I first saw the picture (I felt cold, or I coughed or something else interrupted me).
5) I remembered what I was thinking when I first saw the picture.

Remembering = No.

‘When you answered No to remembering the picture, on what did you base your judgment (check all that apply.)’

1) I just guessed.
2) The picture seemed familiar.
3) I just knew I had seen it before.
4) I did not recognise the other picture.

Participants were given the option of stating an alternative reason. However, only two participants used the option for remember = yes and no participant used this option for remember = no, suggesting that the available responses had covered the majority of their decision reasons.
