# Supplementary material for: Does the Experience of Remembering Differentially Influence the Factual Accuracy of Recognition, and Confidence in Its Accuracy?
Source: J Cogn. 2026 Jan 7;9(1):6. doi: 10.5334/joc.477 (PMC12785665; doi:10.5334/joc.477)
Supplement: Supplementary File 2. — Appendix. Study 1, picture similarity criteria. [file joc-9-1-477-s2.pdf]

**Supplementary file 2: Appendix. Study 1, picture similarity criteria**

In Tulving's original experiment (1981), targets (A) displayed at test represented one half of a colour image appearing across a two-page spread in a popular magazine. 36 A target (old) pictures were shown at study in a target block, together with 12 B non-targets (also representing one half of a two-page colour image, but not used at test). Perceptually similar A' lures had not been seen at study and were the other half of the studied A target images; mnemonically similar B' lures had not been seen at study and were the other half of the studied B non-target images. X lures represented half of a different non-studied two-page spread. None of the lures had been shown at study, i.e., all were "new". All of the A targets had been shown at study , i.e., all were "old".

For our study, the chosen A natural scene targets were paired with A' lures representing the same natural scene , i.e., high in similarity (the images being directly compared at test). The B' lures in the mnemonic similarity condition were chosen to be similar to, but not the same as, a B image shown during the target block (matched features and colours, also being of high similarity to the B non-target). In this case the images cannot be directly compared, and both test images require comparison with memory for studied images of targets and non-targets. The X lures in the dissimilar condition were images that were different from any other image shown in the buffer or target blocks during the study phase, with no comparable studied image stored in memory. In all cases both test-pair images were from the same category, ensuring that the images would be more similar than those from different categories, (e.g., Snodgrass & McCullough, 1986), see Figure 13. All

study and test images and the test picture-pair arrangements are available online as detailed in the data accessibility statement.

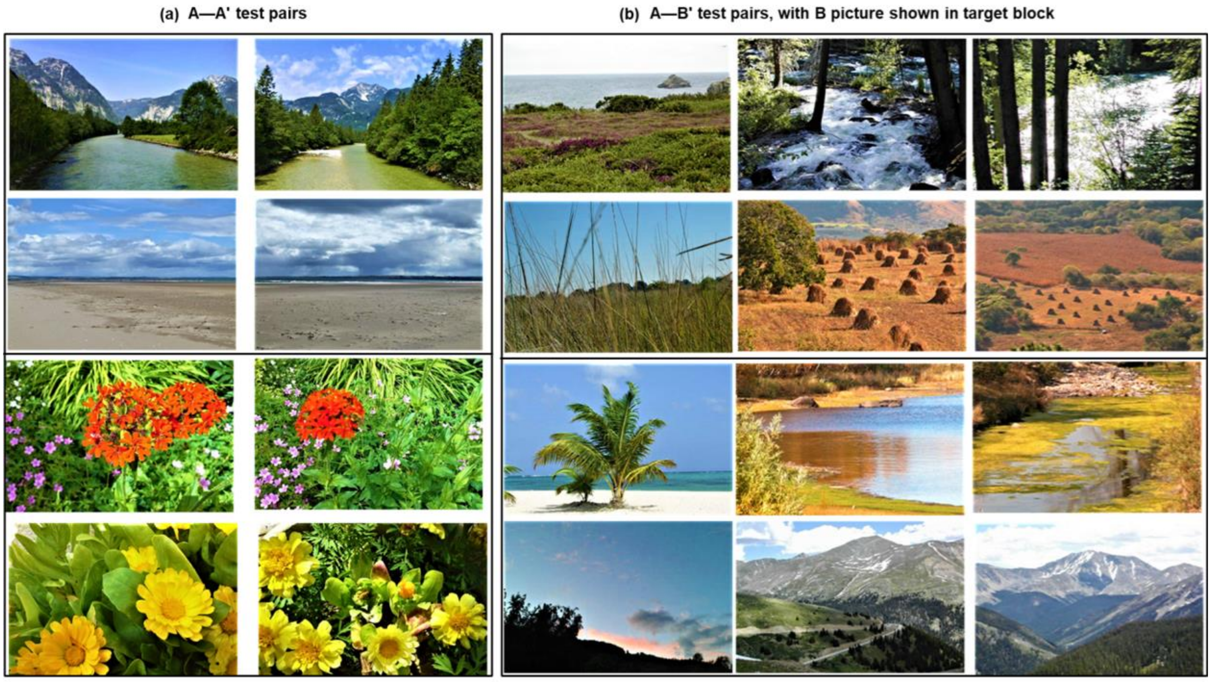


Figure 13. Supplementary file 2: Appendix. Study 1, picture similarity criteria: 2-AFC picture similarity task. (a) perceptually similar A-A' test pairs. (b) mnemonically similar A-B' test pairs. For comparison, the B picture is also illustrated on the far right hand side (shown at study in the target block but not shown at test). [Note. Although the side on which target and lure were presented was randomised, for illustration purposes the target A picture is shown on the left and the lure on the right. The target A picture is taken from the same natural scenes category.as the lure (A', B', and X)].

In other reproductions of the picture similarity task, the A' lures were also drawn from the same scene as the previously studied target, i.e., similar to the previously studied target scene for A-A' test pairs. The B' lure was the other half of a previously studied item In the A-B' condition (Dobbins, Kroll & Liu, 1998). Fandakova et al., (2021) used picture stimuli of various objects, shown with a lure perceptually similar to the target in the A-A' condition. The B' lure was perceptually similar to a different B image (not the target item) shown at study in the A-B' condition. Hembacher and Ghetti, (2017) used stimuli that included colour images of familiar

items, together with images depicting very similar but distinct versions of the same object. Other interpretations have used images of faces (Heathcote et al., 2009), and words presented in different forms at study and test, together with new words not previously seen (Zawadzka et al., 2017).
