# Supplementary material for: Does the Experience of Remembering Differentially Influence the Factual Accuracy of Recognition, and Confidence in Its Accuracy?
Source: J Cogn. 2026 Jan 7;9(1):6. doi: 10.5334/joc.477 (PMC12785665; doi:10.5334/joc.477)
Supplement: Supplementary File 3. — Appendix. Study 2, Instructions to Participants. [file joc-9-1-477-s3.pdf]

Supplementary file 3: Appendix. Study 2, Instructions to Participants.

Vividness

Vividness of memory for the associated cue picture was assessed at test (Figure 10b) by answering “How vividly do you remember the picture” immediately after the image had been re-presented to cue recollection of the cross location (but not while it was still on the screen), and before recollection of the cross location. Response options ranged from not at all vividly, to extremely vividly, on a 0 – 100 horizontal continuous scale.

Accuracy

Accuracy was assessed at test by requiring participants to recall the associated cross location. As shown in Figure 9c, this provides a continuous measure of error for each trial (the difference in degrees between the response and the original location). Location error frequency plots from 0° to ± 180° from the target location, expressed as response frequency across all participants, allow a visual representation of memory performance, e.g., as shown in Figure 10d.

Confidence

Confidence in memory accuracy was assessed at test following recollection of the cross location, but not immediately after rating memory vividness (Figure 9b), with participants answering, “How confident are you that you remembered the right location?” (and not how confident they were that they had remembered the picture). Response options ranged from not at all confident, to very confident, on a horizontal 0 – 100 continuous scale (cf. Harlow & Yonelinas, 2016).
