# Supplementary material for: Does the Experience of Remembering Differentially Influence the Factual Accuracy of Recognition, and Confidence in Its Accuracy?
Source: J Cogn. 2026 Jan 7;9(1):6. doi: 10.5334/joc.477 (PMC12785665; doi:10.5334/joc.477)
Supplement: Supplementary File 4. — Appendix. Study 2, Subsidiary experiments, judging vividness at study. [file joc-9-1-477-s4.pdf]

Supplementary file 4: Appendix. Study 2, Subsidiary experiments, judging vividness at study.

Methods

The identical picture sets were used as in Study 2, Experiments 1 and 2. The relational encoding phases comprised the same picture-cross location pairs as before. The presentation order of the images within each phase (as well as the order of study and test phases) was randomised for each participant using E-prime. Procedures were followed as described above, Figure 10, with the exception that participants for the experiments were instructed to make their vividness judgments during the study phase, immediately after the natural scenes picture was first presented in the relational encoding block (but not when still visible on the screen), answering "How vivid is the picture when you call it to mind?"

Results

Participants

There were 16 participants in the 120-image set group of mean age 27 years (S.D. 5 years); 10 identified as female. There were 16 participants in the 240- image set group, of mean age 22 years (S.D. 4 years); 15 identified as female. The analysis included data from all 32 participants. data were compared by image set.

Variables of interest.

Confidence and accuracy were higher for the 120-image set group, compared to the 240-image set group, with vividness lower, Table 5. Differences in mean values were small, effect sizes were negligible (R^2 change ≤ 0.2%). To summarise, when

vividness of the image was judged when first presented during the study phase, there were no meaningful differences in the variables of interest by image set.

Table 5.

Study 2, subsidiary experiments: variables of interest with vividness of the images judged during the study phase. Data [M (SE)] are compared by image-set using unstandardized B coefficients and R^2 change.

Image Set *(N)*	Variable	*M (SE)*	*F (df)*	Unstandardized B coefficient (*SE*)	*p*-value	R^2 change
120-image (1920)<br>240-image (3840)	Vividness	53.1 (0.5)<br>55.3 (0.4)	12.04 (1,5758)	2.19 (0.63)	< 0.001	0.2%
120-image (1920)<br>240-image (3840)	Confidence	44.9 (0.7)<br>43.2 (0.4)	9.43 (1,5758)	-2.56(0.83)	0.002	0.1%
120-image (1920)<br>240-image (3840)	Accuracy	71.3 (0.7)<br>68.2 (0.5)	13.83 (1,5758)	-3.1 (0.83)	< 0.001	0.2%

Correlational analysis

The relationship between accuracy and confidence was little changed by judging vividness of the images at study, being moderate for the 120-image set group, and weak for the 240-image set group, accounting for less than 20% of the variability in confidence, Table 5. In contrast to judging the remembered vividness of the pictures, the strength of correlation between vividness of the images at study and confidence in source memory accuracy at test was weak for both image sets; < 7% of the variance in confidence can be explained by variability in the perceived vividness of the stimuli when first presented at study, (Table 6, cf. Table 4). Correlations between vividness and source memory accuracy were negligible. As shown in Table 6,

variability in the perceived vividness of the photographs when judged at study explained ≤ 1.4 % of the variance in source memory accuracy.

Table 6.

Study 2, subsidiary experiments: correlations between variables of interest (across all trials) by image set. Data are compared using Pearson's correlation coefficient (r) and variance (R^2).

Image set	*I.V.*	*D.V.*	*N*	Pearson's coefficient (*r*)	*p*-value 2-tailed	R^2 variance
120-image	Accuracy	Confidence	1920	0.451	< 0.001	20.3%
240-image	Accuracy	Confidence	3840	0.39	< 0.001	15.2%
120-image	Vividness	Confidence	1920	0.23	< 0.001	6.9%
240-image	Vividness	Confidence	3840	0.25	< 0.001	5.3%
120-image	Vividness	Accuracy	1920	0.07	< 0.001	0.5%
240-image	Vividness	Accuracy	3840	0.12	< 0.001	1.4%

Discussion

In comparison with Study 2, Table 3, the mean reported vividness of the images at study (240-image set) was the same as was reported at test (Experiment 2), although vividness was rated higher at test when all images were equally vivid (Experiment 1), compared to at study (120-image set). Additionally, mean confidence and accuracy at study (240-image set) were similar to when reported at test (Experiment 2). In sum, images across a full range of vividness ratings were not rated as more vivid when re-presented at test than when initially viewed at study.

In brief, the pattern of vividness judgments made at study differed from that seen at test. Importantly, the correlation between vividness of the images when first viewed at study and accuracy was negligible, and there was only a weak correlation between vividness and confidence. Nonetheless, the correlation between confidence and accuracy (assessed at test) remained moderate and little changed from that seen in Experiments 1 and 2. We conclude therefore that, as instructed, participants in Experiments 1 and 2 were assessing the vividness of their memory (remembered vividness) for the pictures.
